# Human infection with sub-periodic *Brugia* spp. in Gampaha District, Sri Lanka: a threat to filariasis elimination status?

**DOI:** 10.1186/s13071-018-2649-3

**Published:** 2018-01-29

**Authors:** Chandana H. Mallawarachchi, T. G. A. Nilmini Chandrasena, Ranjan Premaratna, S. M. N. S. M. Mallawarachchi, Nilanthi R. de Silva

**Affiliations:** 1 Postgraduate Institute of Medicine, University of Colombo, Colombo, Sri Lanka; 2 Department of Parasitology, Faculty of Medicine, University of Kelaniya, Ragama, Sri Lanka; 3 Department of Medicine, Faculty of Medicine, University of Kelaniya, Ragama, Sri Lanka

**Keywords:** Lymphatic filariasis, Sub-periodic, *Brugia* spp., Brugia rapid test, Sri Lanka

## Abstract

**Background:**

Post-mass drug administration (MDA) surveillance during the lymphatic filariasis (LF) elimination program in Sri Lanka, revealed the re-emergence of brugian filariasis after four decades. This study was done with the objectives of investigating the epidemiology and age-specific vulnerability to infection. Surveillance was done using night blood smears (NBS) and the *Brugia* rapid test (BRT), to detect microfilaria (MF) and anti-*Brugia* IgG4 antibodies in blood samples collected from an age-stratified population enrolled from two high-risk study areas (SA)s, Pubudugama and Wedamulla in the Gampaha District. The periodicity of the re-emergent *Brugia* spp. was characterized by quantitative estimation of MF in blood collected periodically over 24 h using nucleopore-membrane filtration method.

**Results:**

Of 994 participants [Pubudugama 467 (47.9%) and Wedamulla 527 (53%)] screened by NBS, two and zero cases were positive for MF at Pubudugama (MF rate, 0.43) and Wedamulla (MF rate, 0), respectively, with an overall MF rate of 0.2. Of the two MF positives, one participant had a *W. bancrofti* while the other had a *Brugia* spp. infection. Of 984 valid BRT test readings [Pubudugama (*n* = 461) and Wedamulla (*n* = 523)], two and seven were positive for anti-brugia antibodies by BRT at Pubudugama (antibody rate 0.43) and Wedamulla (antibody rate 1.34), respectively, with an overall antibody rate of 0.91. Both MF positives detected from SAs and two of three other *Brugia* spp. MF positives detected at routine surveillance by the National Anti-Filariasis Campaign (AFC) tested negative by the BRT. Association of *Brugia* spp. infections with age were not evident due to the low case numbers. MF was observed in the peripheral circulation throughout the day (subperiodic) with peak counts occurring at 21 h indicating nocturnal sub-periodicity.

**Conclusions:**

There is the low-level persistence of bancroftian filariasis and re-emergence of brugian filariasis in the Gampaha District, Sri Lanka. The periodicity pattern of the re-emergent *Brugia* spp. suggests a zoonotic origin, which causes concern as MDA may not be an effective strategy for control. The importance of continuing surveillance is emphasized in countries that have reached LF elimination targets to sustain programmatic gains.

## Background

Lymphatic filariasis (LF), a neglected tropical disease estimated by the World Health Organization to affect 940 million people in 54 countries, is targeted for elimination by 2020 [1–3]. LF is not a fatal disease, but it can cause significant morbidity. It is the second leading parasitic cause of disability worldwide, estimated to cause 5.549 million disability-adjusted life years (DALYs) [4].

Three species of filarial worms, *Wuchereria bancrofti*, *Brugia malayi* and *Brugia timori* are known to cause lymphatic filariasis in humans [5]. *Wuchereria bancrofti* is responsible for 90% of LF, while *B. malayi* is responsible for most of the remaining infections [6]. *Brugia malayi* is prevalent in Southeast Asia and southwestern India (Kerala) [5]. Both *W. bancrofti* and *B. malayi* infections were prevalent in Sri Lanka in the past [7]. Successful vector control activities targeted at *Mansonia* spp. mosquitoes resulted in complete clearance of brugian filariasis by 1967 [7].

In 2016, Sri Lanka was acknowledged by World Health Organization (WHO) to have eliminated lymphatic filariasis as a public health problem, following five successful rounds of mass drug administration (MDA) conducted in the endemic areas of the country, from 2002 to 2006 [8]. During the national LF elimination programme, only bancroftian filariasis was documented in the endemic areas; brugian filariasis was regarded as eliminated decades previously. However, surveillance activities conducted during the post-elimination phase have found cases of brugian filariasis throughout the endemic region [9–11].

Very little is known of the newly emerged brugian filariasis in Sri Lanka. Therefore, this study aimed to investigate age-specific vulnerability to infection and to characterize the re-emergent species of *Brugia* by its periodicity pattern.

## Methods

### Study area

The study was conducted in the district of Gampaha, owing to the high number of cases reported in the past decade (AFC personal communication). Gampaha District, with 2.3 million inhabitants, is the second most populous district in Sri Lanka and comprises of urban, semi-urban and rural populations in a land area of 1387 km^2^ [12].

**Fig. 1:**
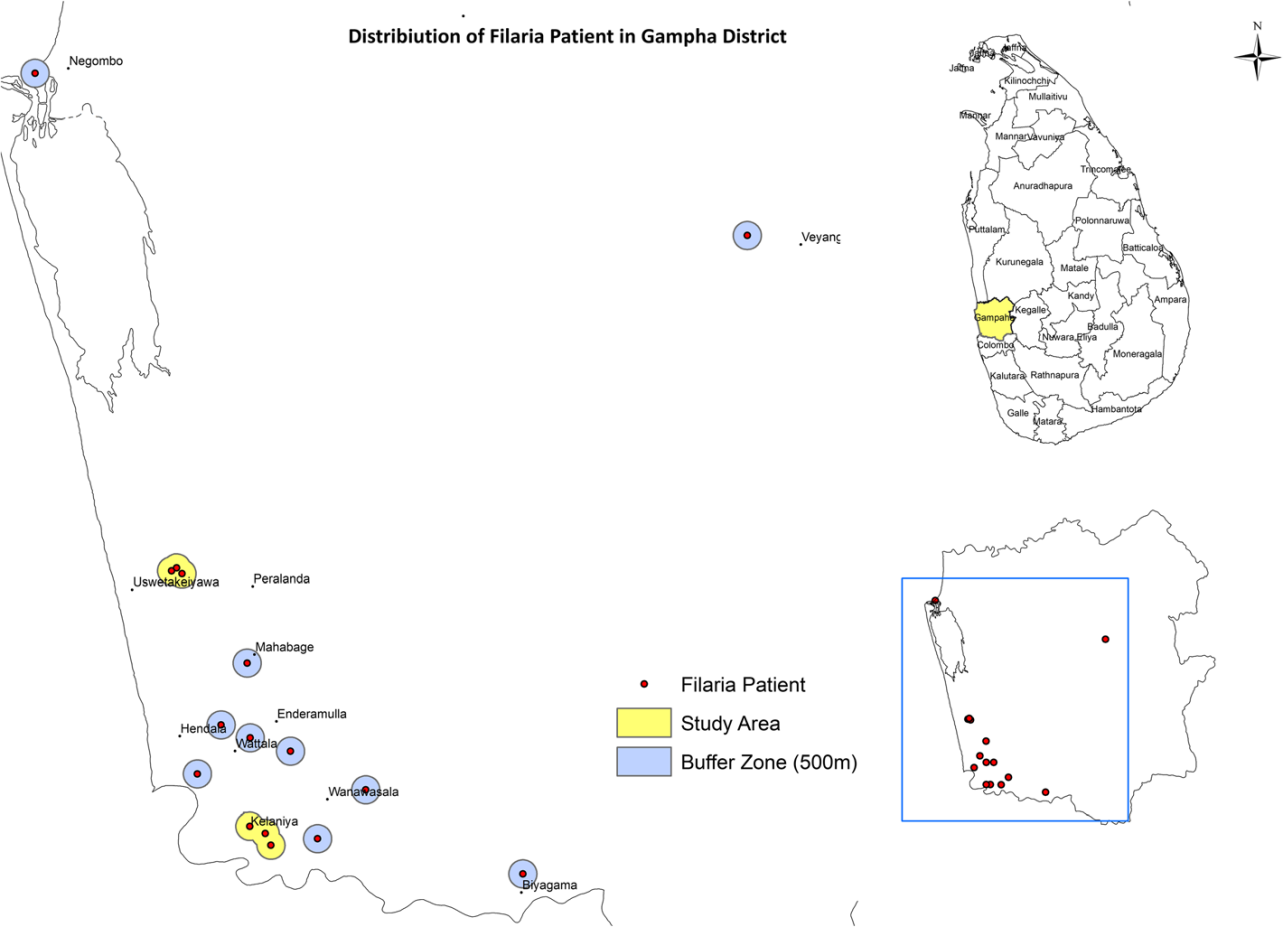
Location of recent past positive cases of Brugian filariasis in Gampaha District in Sri Lanka and 500 m buffer zone of each case. The buffer zones (colored in yellow) were taken as study areas

Study sites were selected based on the occurrence of cases of brugian filariasis in the recent past. All the cases reported from the District of Gampaha were listed, and the GPS coordinates of their households were recorded during site visits, using a handheld GPS monitor (Montana®610 Garmin handheld GPS Receiver), and mapped using ArcGIS 10. A 500 m buffer zone was demarcated around each case. High-risk areas (hot spots) for transmission of brugian filariasis, defined by the occurrence of two or more positive cases within the 500 m buffer zone were selected as study sites. The 500 m radius area of the study locations was decided to encompass the mean flight range of the vector mosquitoes of the genus *Mansonia* (350 m) [13] and an adequate number of households. Two areas that fulfilled the above criteria, Pubudugama and Wedamulla, in Medical Officer of Health areas of Wattala and Kelaniya, respectively, were identified as study sites in this manner. A map indicating the locations of the study sites are given in Fig.1.

### Study population

Criteria for enrolment in the study were residence in a study site for over 1 year and age above 1 year. Households were enrolled by random, systematic sampling method and visited by the surveillance team between 16:00 and 19:30 h for collection of basic demographic data. An age-stratified population of 500 individuals from each study location (total of 1000 individuals) was selected for screening. The age stratification was based on criteria relevant to disease exposure and MDA. The age groups were as follows: 1–5 and 6–10 years of age (born after the conclusion of MDA), 11–20 years (mostly school children, who would have been treated under the MDA programme), 21–50 years (adults who received MDA), 51 years and above (MDA recipients with possible past exposure to *B. malayi* infection). Approximately 100 participants were included from each age group.

### Surveillance and periodicity study

Blood samples were obtained by the house to house screening carried out between 20.00 and 23.00 h during the period December 2016 to June 2017. Two-night blood smears (NBSs) were prepared from each participant (60 μl tri-linear and coin shaped). These were stained with Giemsa and examined microscopically for microfilariae (MF) in two different laboratories: The Department of Parasitology, Faculty of Medicine, University of Kelaniya and the Regional Filariasis Control Unit, Gampaha. Species identification of the MF was based on morphology, namely densely packed nuclei, the presence of two isolated nuclei at the tip of the tail and cephalic space longer than *W. bancrofti* [5].

Each sample of blood was also examined for IgG4 antibodies to *B. malayi* using the Brugia rapid test kit (BRT) from Brugia Rapid, Reszon Diagnostics International, Malaysia. The BRT was performed as per the manufacturer’s instructions in the field, and all positive test results excluding two from non-compliant participants were confirmed by repeat testing. Additional MF positive cases detected at routine surveillance activities of the AFC during this period were also tested for antibody positivity.

The periodicity of a *Brugia* spp. infection detected as described above was studied by quantitative estimation of MF in peripheral blood collected at intervals over 24 h, using the Nucleopore-membrane filtration method [14]. The *Brugia* spp. MF positive case was a male, 14 years of age, who consented to be repeatedly bled. He was admitted to the local hospital, and one milliliter of heparinized venous blood was obtained at 01:00, 03:00, 05:00, 07:00, 09:00, 13:00, 17:00, 21:00 and 23:00 h under aseptic conditions for MF counts.

Data were entered into an Excel file, and age-group specific infection rates (MF and antibody rates) were calculated for each study location. Microfilaria rate is defined as the blood smears positive for microfilaria expressed as a percentage of the smears examined, and the antibody rate is defined as the number of samples found to be antibody-positive by the BRT, as a percentage of the samples examined. The MF rate and antibody rate is equal to the prevalence if a representative sample of the population is screened by the relevant tests; thus, in this study MF rate and antibody rate indicate MF prevalence and antibody prevalence in the study areas. A periodicity curve was prepared by plotting the MF counts against time.

## Results

A total of 994 persons from Pubudugama (*n* = 467, 47.9%) and Wedamulla (*n* = 527, 53%) with a male: female ratio of 1:1.02 from 325 households (153 in Pubudugama and 172 in Wedamulla) were enrolled and screened for LF infection by NBS. The age group specific LF infection rates are as shown in Table 1. Two MF positive cases were detected at Pubudugama (MF rate, 0.43%) and none were found at Wedamulla (MF rate 0), giving an overall MF rate of 0.2. Of the two MF positives, one person (age group 21–50 years) had a *W. bancrofti* infection while the other (age group 11–20 years) had a *Brugia* spp. infection (Table 1).

**Table 1:** Microfilaria and anti-*Brugia* IgG4 antibody positivity according to age groups in Pubudugama and Wedamulla, Sri Lanka

Age group (years)	Number (%) screened		Number (%) positive for LF			
	Pubudugama	Wedamulla	NBS number (%)		BRT Number (%)	
			Pubudugama	Wedamulla	Pubudugama	Wedamulla
1–5	90 (19.3)	88 (16.7)	0	0	0	0
6–10	96 (20.6)	101 (19.2)	0	0	1 (1.04)	1 (0.99)
11–20	91 (19.5)	118 (22.4)	1(Bm) (1.1)	0	1 (1.09)	3 (2.56)
21–50	97 (20.8)	109 (20.7)	1(Wb) (1.03)	0	0	1 (0.92)
> 51	93 (19.9)	111 (21.1)	0	0	0	2 (1.8)
Total	467 (47.9)	527 (53.0)	2 (0.43)	0	2 (0.43)	7 (1.34)

*Abbreviations*: *Bm B. malayi*, *Wb W. bancrofti*, *NBS* night blood smear, *BRT Brugia* rapid test

The BRT was done on 991 participants with valid test readings obtained in 984 (461 in Pubudugama, and 523 in Wedamulla). Nine participants [two in Pubudugama, (antibody rate 0.43%) and seven in Wedamulla (antibody rate 1.34%)] were positive for anti-Brugia IgG4 antibodies (overall antibody rate 0.91) (Table 1). Both MF positive individuals were negative for antibodies by the BRT. The BRT was done on three other MF (*Brugia* spp.) positive cases (all < 5 years) detected by the AFC during routine surveillance activities and only one of them tested positive for antibodies.

**Fig. 2:**
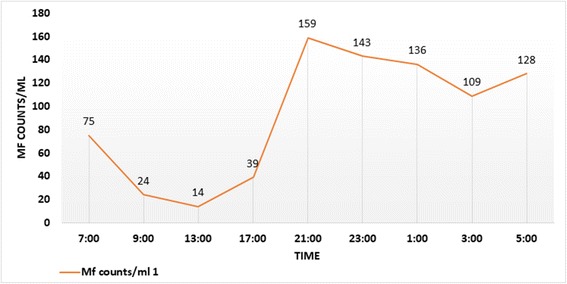
Throughout the day MF were positive in the peripheral blood indicating nocturnal sub-periodicity

In MF positive individual studied for periodicity (Fig. 2), MF was observed in the peripheral circulation throughout the day (subperiodic) with peak counts observed at 21.00 h indicating a nocturnally-subperiodic parasite species.

## Discussion

The overall MF and anti-Brugia antibody rates observed in our study population were 0.20 and 0.91, respectively, which is below the thresholds used to define LF endemicity by WHO (prevalence of MF and filarial-specific antibodies 1% and 2%, respectively) [15]. A previous survey for anti-*Brugia* antibodies among a paediatric population (mean age 8.5 years) in Pubudugama reported a slightly higher antibody rate of 1.6 [9]. Although the present survey was restricted to Gampaha District, cases of brugian filariasis are increasingly detected at routine night blood surveys of the endemic region (four and ten MF positive cases in 2015 and 2016, respectively) [11, 16].

Persistent transmission of LF, after cessation of mass chemotherapy based intervention programs, has been described in Pondicherry (20 years after cessation of a DEC-fortified salt program with mop-up MDA), in Ghana after 14 years of MDA and in American Samoa 8 years after cessation of MDA [17–19]. Although, low MF rates of 0.6–0.7 were described by Ramiah et al. [17] as safe, with very little likelihood of recrudescence, vigilance is critical at this point to ensure that programmatic gains are sustained.

Two strains of *B. malayi* have been recognized based on the periodicity patterns of MF [20, 21]. Humans are the only known definitive hosts of the nocturnally periodic form of *B. malayi*, but the nocturnally subperiodic form shows little host specificity with zoonotic reservoirs in primates and feline species (palm civet cats, wild cats and domestic cats) and cause infections in humans as well [22–24]. The *B. malayi* that was prevalent in Sri Lanka in the past was the nocturnally periodic form [25]. Subperiodic *B. malayi* has been reported in Southeast Asia (Indonesia, Peninsular Malaysia, the Philippines and Thailand) where it has been described as an important zoonosis with animal-to-man and even man-to-man transmission [22, 26]*.*

*Brugia pahangi,* a parasite of cats and dogs, is another zoonotic filarial parasite that exhibits nocturnal periodicity. Reports of natural *B. pahangi* infections in humans are rare, perhaps because of difficulty in distinguishing between the microfilariae of *B. pahangi* and *B. malayi*, unless special staining techniques are employed [22]. Recently, several cases of symptomatic *B. pahangi* infections were reported from Malaysia where the identity of the infecting species was established by PCR [27].

The control of subperiodic *B. malayi* infection poses a great problem as mass treatment of the human population with antifilarials, so effective for periodic *B. malayi*, and *W. bancrofti* does not suffice because the large animal reservoir remains untouched [22, 26]. Thus the re-emergence of Brugian filariasis infections in Sri Lanka after several decades, is a cause for concern, especially because the nocturnal subperiodicity of the MF suggests a zoonotic infection. Moreover, a recent survey in the Western Province of Sri Lanka has demonstrated a canine reservoir of *B. malayi* infection [28].

Association of *Brugia* spp. infections with age was not evident in the present survey due the low number of cases detected (one MF and 9 antibody positives, *n* = 10). However, the majority of cases (70%) in the present study occurred among the under 20-year age group.

The rapid diagnostic used in this survey, BRT detects filaria-specific IgG4 antibodies to recombinant antigen BmR1 of *Brugia malayi.* Anti-filarial IgG4 levels have considered being a good indicator of active infection since levels are elevated in active filariasis [29–31] and decline post-treatment [32, 33]. Thus, detection of anti-filarial IgG4 antibodies has been used for epidemiological assessment of filariasis [34].

In the present survey, BRT was positive in only one of four microfilaraemia cases that were positive by NBS, whereas nine BRT positive individuals were MF negative. A multicenter laboratory evaluation of BRT for detection of brugian filariasis have reported sensitivities of > 95% with 100% specificity [35]. However, a field validation of BRT done in Malaysia reported a lower sensitivity (87%) as compared to microscopy of NBS, but BRT detected about ten times more cases than microscopy [36]. The latter findings are comparable with the results of the present study. The lower sensitivity of BRT in the field than in laboratory evaluations was attributed to the small number of microfilaremic individuals and difficulties encountered in performing the test in remote villages of Malaysia [36]. The small number of microfilaraemia patients in the present study may be the explanation for the lower sensitivity of the BRT for detecting them. Perhaps, genetic predisposition to infection and capacity to mount antibody responses are dissociated, and this only becomes noticeable when prevalence is low.

The periodicity data obtained in this study cannot be generalized to all the re-emergent *Brugia* infections in Sri Lanka, as only a single case was studied due to the paucity of eligible cases. This limitation should be addressed by further periodicity studies and establishment of species identity by molecular methods.

## Conclusions

There was the low-level persistence of bancroftian filariasis and re-emergence of brugian filariasis in Gampaha District, Sri Lanka. One case of re-emergent *Brugia* species infection was found to exhibit nocturnal sub-periodicity, suggesting a probable zoonotic origin. This report highlights the implications of an untouched zoonotic reservoir of *B. malayi* infection coupled with minimal on-going vector control activities in Sri Lanka, which could threaten the successes achieved in LF control.

## Abbreviations

AFC: Anti-filariasis campaign
BRT: *Brugia* rapid test
DALYs: Disability-adjusted life years
LF: Lymphatic filariasis
MDA: Mass drug administration
MF: Microfilaria
NBS: Night blood smears
PCR: Polymerase chain reaction
SA: Study areas
WHO: World Health Organization

## References

[1] WHO. Lymphatic filariasis. World Health Organ Fact sheets 2017;102 www.who.int/mediacentre/factsheets/fs 102/en/ Accessed 10 Oct 2017.

[2] Ottesen E. The global programme to eliminate lymphatic filariasis. Trop Med Int Health. 2000;5:591–4. 10.1046/j.1365-3156.2000.00620.x 11044272

[3] WHO (2010). Global programme to eliminate lymphatic filariasis. Progress report 2000–2009 and strategic plan 2010–2020.

[4] Fenwick A (2012). The global burden of neglected tropical diseases. Public Health.

[5] Simonsen PE, Fischer PU, Hoerauf A, Weil GJ, Farrar J, Hotez PJ, Junghanss T, Kang G, Lalloo D, White NJ (2014). The filariases. Manson’s tropical diseases.

[6] WHO (1992). Lymphatic filariasis; the disease and its control. Fifth report of the WHO expert committee on Filariasis. World Health Organ Tech Rep Ser.

[7] Schweinfurth U (1983). Filarial diseases in Ceylon: a geographic and historical analysis. Ecol Dis.

[8] WHO Country Office for Sri Lanka. WHO Officially declares Sri Lanka Filariasis free. Colombo: World Health Organization; 2016. http://www.searo.who.int/srilanka/documents/WHO_officially_declares_Sri Lanka_filariasis_ free/en/ Accessed on 06 Oct 2017.

[9] Chandrasena TGAN, Premaratna R, Samarasekera DS, de Silva NR. Surveillance for transmission of lymphatic filariasis in Colombo and Gampaha districts of Sri Lanka following mass drug administration. Trans R Soc Trop Med Hyg. 2016;5:620–2. 10.1093/trstmh/trw067 27816936

[10] Fisher PE, Liyanage T, Rao RU, Weil GJ. Molecular characterization of re-emergent *Brugia malayi* in Sri Lanka. Final Program 57^th^ ASTMH; 2008 10.4269/ajtmh.2008.79.1a. page 132 accessed 8 Jan 2017.

[11] Anti Filariasis Campaign. Annual Statistical Bulletin. 2015. Anti Filariasis Campaign - Ministry of Health and indigenous Medicine Sri Lanka. Sri Lanka: Ministry of Health and Indigenous Medicine; 2015.

[12] Department of Census and Statistics Sri Lanka. Statistics Dept.; 2012. www.statistics.gov.lk/ Accessed on 10 Oct 2017.

[13] Gass RF, Deesin T, Sucharit S, Surathin K, Vutikes S (1983). Dispersal and flight range studies on *Mansonia annulata*, *Ma. indiana*, and *Ma. uniformis* (Diptera: Culicidae) in southern Thailand. J Med Entomol.

[14] Garcia LS, Buckner DA. Diagnostic Medical Parasitology: Procedures for Detecting Blood Parasites. 2nd ed. Washington DC: American Society for Microbiology; 1993. p. 591.

[15] World Health Organization. Monitoring and epidemiological assessment of mass drug administration in the global programme to eliminate lymphatic filariasis: a manual for national elimination programmes. Geneva: WHO; 2011.

[16] Anti Filariasis Campaign. Annual Statistical Bulletin. Anti Filariasis Campaign - Ministry of Health and Indigenous Medicine, Sri Lanka. Sri Lanka: Ministry of Health and Indigenous Medicine; 2016.

[17] Ramaiah KD, Thiruvengadam B, Vanamail P, Subramanian S, Gunasekaran S, Nilamani N, et al. Prolonged persistence of residual *Wuchereria bancrofti* infection after cessation of diethylcarbamazine-fortified salt programme. Trop Med Int Health. 2009;14(8):870–6. 10.1111/j.1365-3156.2009.02307.x 19552662

[18] Biritwun NK, Yikpotey P, Marfo BK, Odoom S, Mensah EO, Aseidu O (2016). Persistent ‘hot spots’ of lymphatic filariasis microfilaraemia despite 14 years of mass drug administration in Ghana. Trans R Soc Trop Med Hyg.

[19] Lau CL, Sheridan S, Ryan S, Roineau M, Andreosso A, Fuimaono S (2017). Detecting andconfirming residual hotspots of lymphatic filariasis transmission in American Samoa 8 years after stopping mass drug administration. PLoS Negl Trop Dis.

[20] Sasa M. A review on classification and geographic distribution on brugian filariasis. Joint WPRO/SEARO Working Group on Brugian Filariasis. Geneva: World Health Organizatioin; 1979.

[21] Partono F (1987). Purnomo. Periodicity studies of *Brugia malayi* in Indonesia: recent findings and a modified classification of the parasite. Trans R Soc Trop Med and Hyg.

[22] Dissanaike AS (1979). Zoonotic aspects of filarial infections in man. Bull World Health Org.

[23] Kanjanopas K, Choochote W, Jitpakdi A, Suvannadabba S, Loymak S, Chungpivat S, Nithiuthai S (2001). *Brugia malayi* in a naturally infected cat from Narathiwat Province, southern Thailand. Southeast Asian J Trop Med Public Health.

[24] Chansiri K, Tejangkura T, Kwaosak P, Sarataphan N, Phantana S, Sukhumsirichart W (2002). PCR based method for identification of zoonotic *Brugia malayi* microfilariae in domestic cats. Mol Cell Probes.

[25] McNulty SN, Mitreva M, Weil GJ, Fischer PU (2013). Inter and intra-specific diversity of parasites that cause lymphatic filariasis. Infect Genet Evol.

[26] WHO. Lymphatic filariasis: fourth report of the WHO expert committee on filariasis. Geneva: World Health Organization Technical Report Series; 1984. 6435317

[27] Tan LH, Fong MY, Mahmud R, Muslim A, Lau YL, Kamarulzaman A (2011). Zoonotic *Brugia pahangi* filariasis in a suburbia of Kuala Lumpur City, Malaysia. Parasitol Int.

[28] Kuruppu KAAS, Wickramasinghe S, Samarasekera SD, Wijegunawardana NDAD (2014). Detection of human filarial parasite *Brugia malayi* in dogs in Sri Lanka. SLJCR.

[29] Ottesen EA, Skvaril LF, Tripathy S, Pointdexter RW, Hussain R (1985). Prominence of IgG4 in the IgG response to human filariasis. J Immunol.

[30] Kurniawan A, Yazdanbakhsh M, van Ree RR, Aalberse R, Selkirk ME, Partono F (1993). Differential expression of IgE and IgG4 specific antibody responses in asymptomatic and chronic human filariasis. J Immunol.

[31] Rahman N, Anuar AK, Ariff RH, Zurainee MN, A’shikin AN, Fadzillah A, et al. Use of antifilarial IgG4-ELISA to detect *Brugia malayi* infection in an endemic area of Malaysia. Trop Med Int Health. 1998;3(3):144–8. 10.1046/j.1365-3156.1998.00229.x 9593356

[32] Wamae CN, Roberts JM, Eberhard ML, Lammie PJ (1992). Kinetics of circulating IgG4 after diethylcarbamazine and ivermectin treatment of bancroftian filariasis. J Infect Dis.

[33] Kurniawan A, Atkinson R, Sartono E, Partono F, Yazdanbakhsh M, Maizels RM (1995). Differential decline in filarial specific IgG1, IgG4 and IgE antibodies in *Brugia malayi* infected patients after diethylcarbamazine therapy. J Infect Dis.

[34] Haarbrink M, Terhell A, Abadi K, van Beers S, Asri M, de Madeiros F, Yazdanbakhsh M. Anti-filarial IgG4 in men and women living in *Brugia malayi* endemic areas. Trop Med Int Health. 1999;4:93–7. 10.1046/j.1365-3156.1999.00367.x 10206262

[35] Rahmah N, Shenoy RK, Nutman TB, Weiss N, Gilmour K, Maizels RM, et al. Multicentre laboratory evaluation of *Brugia* rapid dipstick test for detection of brugian filariasis. Trop Med Int Health. 2003;8(10):895–900. 10.1046/j.1365-3156.2003.01102.x 14516300

[36] Jamail M, Andrew K, Junaidi D, Krishnan AK, Faizal M, Rahmah N. Field validation of sensitivity and specificity of rapid test for detection of *Brugia malayi* infection. Trop Med Int Health. 2005;10(1):99–104. 10.1111/j.1365-3156.2004.01334.x 15655019

